# High Conductivity and Thermoelectric Power Factor in p-Type MoS2 Nanosheets

**DOI:** 10.1021/acsaem.4c02932

**Published:** 2025-02-05

**Authors:** Inés Durán, Carlos Bueno-Blanco, Jorge Rodríguez-Muro, Mario Martinez, Elizabeth Champa-Bujaico, Patricia Cancho García, Der-Yuh Lin, Antonio Marti, Elisa Antolin, Simon A. Svatek

**Affiliations:** 1 Instituto de Energía Solar, Universidad Politécnica de Madrid, Avenida Complutense 30, Madrid 28040, Spain; 2 Department of Electronics Engineering, National Changhua University of Education, Changhua 50007, Taiwan

**Keywords:** layered materials, transition metal dichalcogenides, doping, thermoelectricity, electrical conductivity

## Abstract

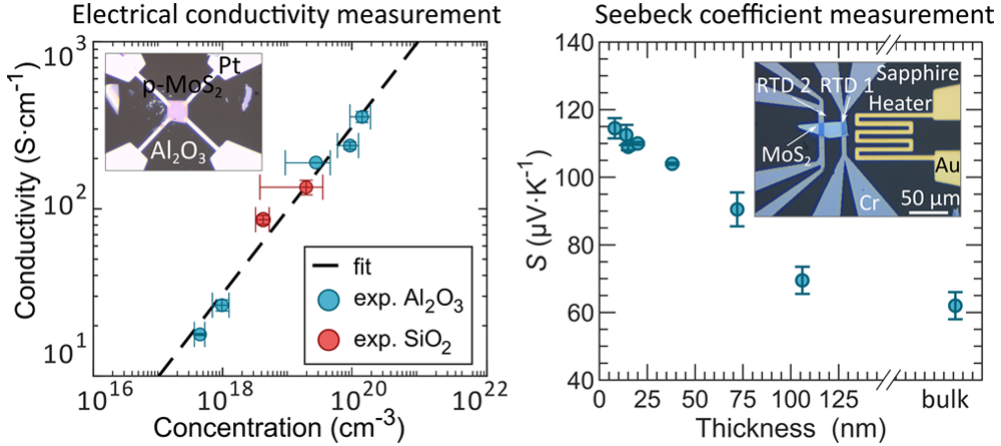

Transition metal dichalcogenides, particularly Nb-doped MoS_2_, present unique electronic and thermoelectric properties that make them promising candidates for a variety of applications, including photovoltaic cells and thermoelectric devices. Here, we investigate the influence of controlled substitutional doping on the electrical conductivity and thermoelectric performance of MoS_2_ as a function of crystal thickness. We report an exceptional bulk conductivity of up to 360 ± 30 S cm^–1^ and a peak power factor of 370 ± 80 μW m^–1^ K^–2^ at room temperature. Our findings reveal that the interplay between doping concentration and thickness can decouple the Seebeck coefficient from electrical conductivity, overcoming the typical trade-off observed in conventional materials. This research highlights the role of surface effects and depletion regions in p-type transition metal dichalcogenides, providing a pathway for developing efficient bipolar thermoelectric devices. The stability and tunability of p-type doping in MoS_2_ also suggest potential applications in microscale cooling, thermal sensors, and photovoltaic systems.

## Introduction

Transition metal dichalcogenides (TMDs) exhibit a range of properties that make them attractive for electronic applications such as high optical absorption coefficients, mechanical flexibility, and potential for large-scale growth and processing.^1−6^ Significant effort has been devoted to develop TMD-based devices with notable successes in areas such as photodetectors, and gas sensors have been achieved with performances that overcome other technologies in terms of response time and responsivity.^7,8^ There have also been attempts at fabricating bipolar devices such as solar cells^9−12^ and thermoelectric converters^13−15^ with localized electrostatic gating or surface doping; however, these devices are characterized by low performance due to the absence of a stable and controllable doping. To date, bipolar devices made of TMDs have not outperformed other established technologies and the reported efficiencies have been far below that of alternative hybrid^16^ or standard silicon technology. Achieving competitive performance in bipolar TMD devices requires the development of stable and controlled doping methods, as well as a deeper understanding of the relationship between crystal thickness and device performance.

In a classical semiconductor device, there is a clear distinction between the bulk and the surface of the electronically active material. Bulk properties, such as conductivity, doping concentration, band gap energy, dielectric constant, and absorption coefficient govern the device operation. However, surface properties may vary due to defects such as dangling bonds often leading to higher recombination rates. The proximity to the substrate or capping layer can also affect the surface, causing either accumulation or depletion of carriers in certain regions. Numerous techniques minimize surface effects in conventional semiconductors, enabling a device purely determined by bulk properties. However, producing ultrathin devices with thicknesses in the range of nm or tens of nm presents significant challenges. In such cases, the surface constitutes a substantial proportion of the device, resulting in the surface regions predominantly governing the device operation. This phenomenon is particularly relevant in studies of mono- or few-layer TMDs. For example, in refs (17−20), reported electron concentrations in 2D are in the range of 10^13^ to 10^15^ cm^–2^ whereas the bulk concentration is in the order of 10^17^ cm^–3^. These high concentrations in 2D have been attributed to the formation of sulfur vacancies that lead to electron accumulation.^21,22^ This phenomenon occurs at the surface, and, consequently, its significance diminishes as the material thickness increases. Several implications arise from this; for example, the apparent conductivity of an n-type crystal increases as the thickness decreases. It would be interesting to disentangle surface effects from the bulk conductivity. This is particularly interesting for p-type material, in which electron accumulation results in depletion rather than an increase in conductance.

Moreover, TMDs,^23^ particularly MoS_2_, are promising alternatives to commercial thermoelectric materials. Traditional thermoelectric materials usually comprise toxic, rigid, heavy, or scarce elements such as Bi, Te, Co, and Pb, prompting the search for more sustainable alternatives. Often, the thermoelectric figure of merit ZT is used to assess the efficiency of a candidate material; however, for certain applications, such as cooling, efficiency may be less critical than the total achievable power. In such applications, the most relevant parameter is the thermoelectric power factor.^24,25^ Established thermoelectric materials exhibit power factors ranging from 100 to 10,000 μW m^–1^ K^–2^ at room temperature.^26−28^ In its bulk state, MoS_2_ demonstrates a moderate power factor of 25 μW m^–1^ K^–2^, which can be enhanced through several strategies. Heavy doping, for instance, elevates values up to 73 μW m^–1^ K^–2^,^29^ while the inclusion of carbon nanotubes (CNTs) shows an increase of the power factor to 120 μW m^–1^ K^–2^.^15^ Furthermore, by introducing VMo_2_S_4_ as a secondary phase via vanadium doping (p-type), the power factor increases to a peak value of 438.95 μW m^–1^ K^–2^ at 1000 K for MoS_2_-5%V.^30^ Incorporating Mo_2_S_3_^31^ or MoO_3_^32^ into MoS_2_ also improves the power factor, reaching 60 μW m^–1^ K^–2^ at 460 K or 411.43 μW m^–1^ K^–2^ at 600 K, respectively.

MoS_2_, as a van der Waals material, maintains mechanical stability down to a monolayer thickness. At a thickness of one to a few monolayers, the thermoelectric power factor is significantly enhanced due to large conduction band effective masses, exceeding 5000 μW m^–1^ K^–2^.^33^ However, this enhancement requires three-terminal devices and precise thickness control down to a monolayer, both of which are incompatible with cost-effective, large-scale production methods. In practice, low-cost deposition methods for MoS_2_, and other TMDs, such as inkjet printing and electrophoretic deposition, typically produce thicker layers in the range of 5–100 nm.^34−39^ References (34−39) also contain further details on scalable deposition methods. This study explores the transition from few-layer to bulk properties in MoS_2_. In this intermediate thickness range, the crystals retain advantages characteristic of mono- and bilayers, such as flexibility and lightweight properties, while simultaneously acquiring benefits associated with bulk materials, including reduced sensitivity to environmental effects. Despite its potential, this thickness range has been widely unexplored for thermoelectric applications. This may be because the band structure of the monolayer is significantly different from the bilayer and further changes in band structure are less and less significant with increasing thickness.^33,40−42^ This trend is also evident in the minor variations observed in photoluminescence beyond 3 nm thickness.^42^

In this work, we study MoS_2_ with Nb doping (MoS_2_:Nb) that induces p-type charge carrier transport, as a model system to quantify how substitutional doping impacts the size of depleted regions. Van der Pauw measurements reveal the interplay between doping concentration and size of depleted regions and quantify bulk properties, namely, mobility and electrical conductivity. Interestingly, we find very high bulk electrical conductivities of up to ∼400 S cm^–1^. Furthermore, we characterize the thermoelectrical properties of MoS_2_:Nb. We determine the effective mass and the dependence of the in-plane Seebeck coefficient as a function of the doping concentration. As the material thickness decreases, we observe a decoupling of the Seebeck coefficient and the electrical conductivity, similar to the behavior of two-dimensional electron gases.^43^ This decoupling leads to an increase in power factor when the thickness is reduced. At a thickness of 8 nm and a carrier concentration of 10^20^ cm^–3^, we observe a room temperature power factor of 370 ± 80 μW m^–1^ K^–2^.

## Results and Discussion

**Figure 1:**
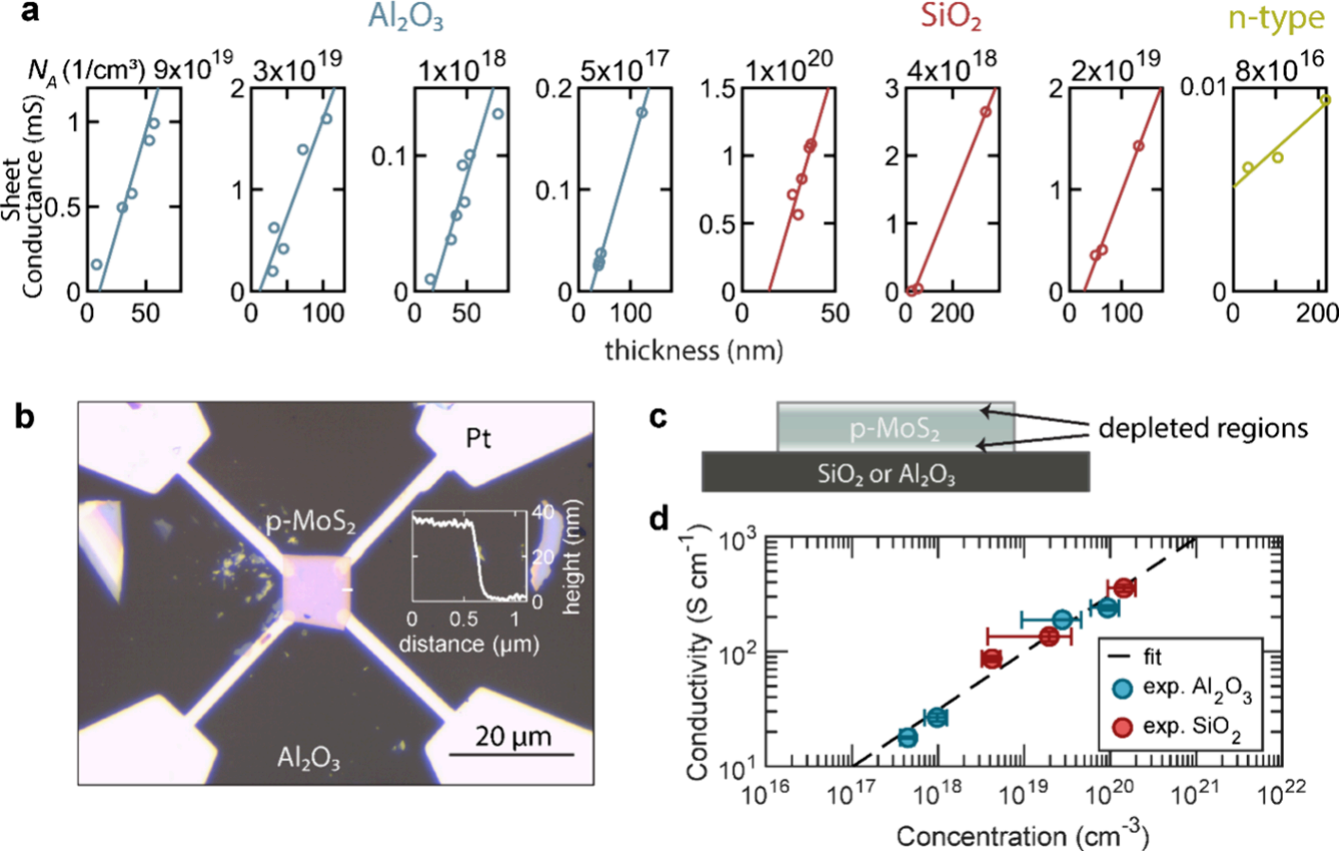
Measurement of conductivity and depleted thicknesses in p-type MoS_2_:Nb. (a) Conductance exhibits a linear dependence on thickness. Linear fits cross the thickness axis at a positive value indicating the total thickness of the depleted regions. For pristine material (unintentionally n-doped) on SiO_2_, the opposite is observed, indicating surface charge accumulation. (b) Optical microscopy image of a typical device to measure electrical conductivity and carrier concentration of exfoliated MoS_2_:Nb using the van der Pauw method. Inset: AFM profile to determine crystal thickness. (c) Section of the substrate on which the MoS_2_ crystals are deposited, either SiO_2_ or Al_2_O_3_. Depleted regions reduce the effective electrical material thickness. (d) Electrical conductivity against carrier concentration. The colors indicate the substrate: SiO_2_ in red and Al_2_O_3_ in blue.

Figure 1a shows the sheet conductance of MoS_2_:Nb as a function of thickness for different carrier concentrations. Thin laminae were mechanically exfoliated from bulk crystals grown using the chemical transport method,^44,45^ with Nb doping level between 0.1 and 1% with respect to the density of host Mo sites. The growth methods have been described previously.^44,45^ Afterward the exfoliated crystal was transferred onto prepatterned electrodes supported by Al_2_O_3_ or SiO_2_. A typical device is shown in Figure 1b. The crystal thickness was determined by atomic force microscopy (AFM), see inset. The sheet conductances in Figure 1a were measured using the Van der Pauw method, and the carrier concentration *N* was determined from Hall effect measurements performed on the same samples. A geometric correction was applied to account for the nonideality of the contacts^46−48^ (see the Supporting Information). For any carrier concentration, the sheet conductance increases linearly with thickness as shown in Figure 1a and higher slopes correspond to higher carrier concentrations.

For a conventional 3D semiconductor, one would expect the conductance to vanish as the thickness approaches zero. Contrarily, here the linear fit consistently crosses the *x*-axis at a positive value. This offset indicates that a part of the material is depleted from carriers and only a reduced crystal thickness contributes to the conductance. If pristine MoS_2_ (n-type) is deposited on SiO_2_, we find the opposite behavior: the linear fit in the conductance/thickness graph crosses the *y*-axis indicating residual conductance at diminishing material thickness.

The plots in Figure 1a allow for the distinction between surface and bulk electronic contributions: the slope is proportional to the bulk conductivity, and the offset in the horizontal axis indicates the thickness of the carrier depleted region at the surface (schematically shown in Figure 1c). Notably, the depleted thickness varies with the substrate, as further discussed below. We attribute the depleted regions to surface electron accumulation which causes exorbitant doping concentrations of the order of 10^13^ to 10^15^ cm^–2^ in pristine MoS_2_ monolayers.^49−52^ Electrons gradually accumulate at the surfaces due to desulfurization and acceptor-like surface states located in the bandgap.^52^ In n-type material, this effect contributes to conduction. Here, in p-type material, it fills holes in the regions closest to air and the substrate.

The electrical conductivity extracted from the linear fit is shown in Figure 1d. Since the conductivity is a bulk property, it does not depend on the substrate and all data points align well with a single fit. This implies that the (bulk) conductivity is constant for a given doping concentration. The carrier concentration, shown on the *x*-axis, is also a bulk property and it has been extracted from Hall measurements performed on the same samples with van der Waals geometry. We highlight that, although surface electron accumulation depletes p-type material, p-type MoS_2_ still achieves elevated conductivity values comparable to the highest reported for thin n-type MoS_2_. Even at 10^17^ cm^–3^ doping concentrations, the conductivity surpasses bulk n-type MoS_2_ by two orders of magnitude.^53^ While pristine MoS_2_ commonly exhibits a 2H stacking, Nb doping causes a structural transformation to the 3R-MoS_2_ phase.^45^ 3R-MoS_2_ recently attracted much interest because it facilitates electrical contacts,^54^ it is promising for optoelectronic applications,^12,44^ and it has a low thermal conductivity.^55^ Both polytypes have trigonal prismatic coordination of the metal, but they have different crystal lattice symmetries, 2H is hexagonal while 3R is rhombohedral. Our Nb-doped samples have concentrations in the range between 10^17^ and just above 10^20^ cm^–3^ with the highest doping level being degenerate. This aligns with tests showing that it produces ohmic contacts with various metals with different work functions: Cr, Pt, and Au (see Supporting Information).

**Figure 2:**
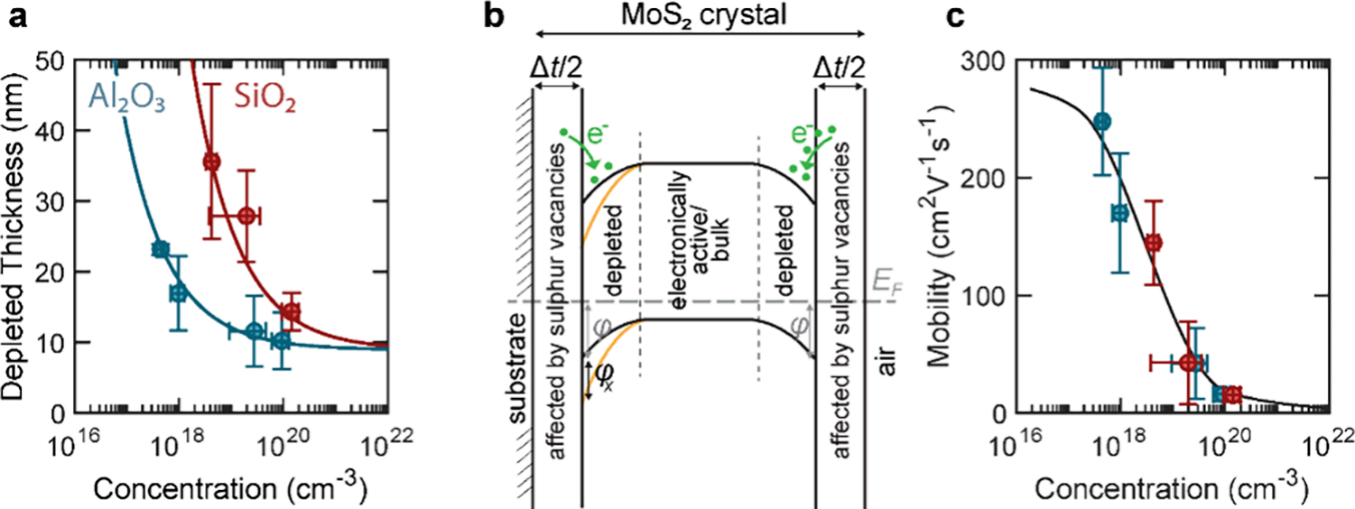
Surface and bulk electronic properties of MoS_2_:Nb. (a) Experimentally determined depleted thickness against carrier concentration. The solid lines show a fit using the total depletion approximation. (b) Schematic of band bending in proximity to the substrate. (c) Mobility measured by the van der Pauw method against carrier concentration (solid line is a guide to the eye).

The thickness of the depleted regions depends on the carrier concentration such as that shown in Figure 2a. We find that the substrate markedly modifies the depleted regions, resulting in two clearly distinct sets of data observed between MoS_2_ crystals on SiO_2_ and on Al_2_O_3_. Devices on SiO_2_ show a strong increase of depleted thickness when the carrier concentration reduces to 10^19^ cm^–3^. By contrast, such increase in depleted thickness occurs in devices on Al_2_O_3_ at lower carrier concentrations. On both substrates at high concentrations of ∼10^20^ cm^–3^, the depleted thickness has a value of the order of 10 nm. We attribute this behavior to charge redistribution within the crystal because of the occurrence of sulfur vacancies at the surfaces and negative charges transferred from the surface. To quantify this, we calculated the depleted thickness *t* considering the band structure shown in Figure 2b. Due to sulfur vacancies, regions of a total thickness of Δ*t* do not conduct charge carriers. To obey charge neutrality, the additional free electrons that occur through the absence of sulfur atoms redistribute into the inner crystal and cause depleted regions. They can be modeled by considering the crystal in contact with an electrochemical potential offset from that of MoS_2_:Nb by φ. Additionally, the substrate on the bottom side of the crystal introduces an additional offset φ_*x*_. The total extension of the depleted thickness can thus be estimated using the total depletion approximation,^56^
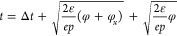
1where ε is the permittivity, *e* is the electron charge, and *p* is the doping concentration. Fitting this model to the data, we determine Δ*t* ≅ 7 nm, φ = 0.02 V, φ_SiO_2__ = 1 V, φ_Al_2_O_3__ = 0 V. Considering the modeling results, we can summarize the effects of the different substrates on the thickness of the depleted regions. At concentrations above 10^20^ cm^–3^, the depleted thickness is similar for both substrates and approaches the thickness of the regions directly affected by sulfur vacancies. At lower concentrations, SiO_2_ introduces a larger amount of band bending since there is a higher amount of accumulated charge, possibly due to higher hydrophilicity, a higher number of polar adsorbates such as water, or a larger amount of charged impurities. The Al_2_O_3_ substrates introduce no significant additional band bending.

In contrast to the depleted thickness, which is a surface property, the carrier mobility is a bulk property that is found to be independent of the surface. The mobility against carrier concentration is shown in Figure 2c. The carrier concentration has been obtained from Hall measurements, and the mobility has been extracted from the expression μ_h_ = *R*_S_/*en*_s_, where *R*_S_ is the sheet mobility and *n*_s_ is the sheet carrier density. Indeed, we find that all points fit onto a characteristic S-shaped curve independently of the substrate. Also, the electrical conductivity in Figure 1d is independent of the substrate. In summary, we can apply the classical picture of the bulk and surface to provide a comprehensive picture of conductivity, mobility, carrier concentration, and depleted regions for this TMD.

Our results clearly indicate that the electronic thickness of p-type MoS_2_ is different from the mechanical material thickness. This implies a decrease in bulk contribution to electron transport in thinner crystals. Here, we extend the study to thermoelectrical properties, since lowering the dimensions of thermoelectric materials is typically accompanied by an increase in performance,^57,58^ and in line with the results above, this improvement is expected to be reachable at thicknesses well above 10 nm. The thermoelectric performance of a device is frequently assessed using the figure of merit, ZT. However, for numerous applications, such as cooling, efficiency is less critical than the total obtainable power. In these cases, the most important parameter is the thermoelectric power factor,^24,25^ which is the product of the squared Seebeck coefficient, *S,* and the electrical conductivity, σ.

**Figure 3:**
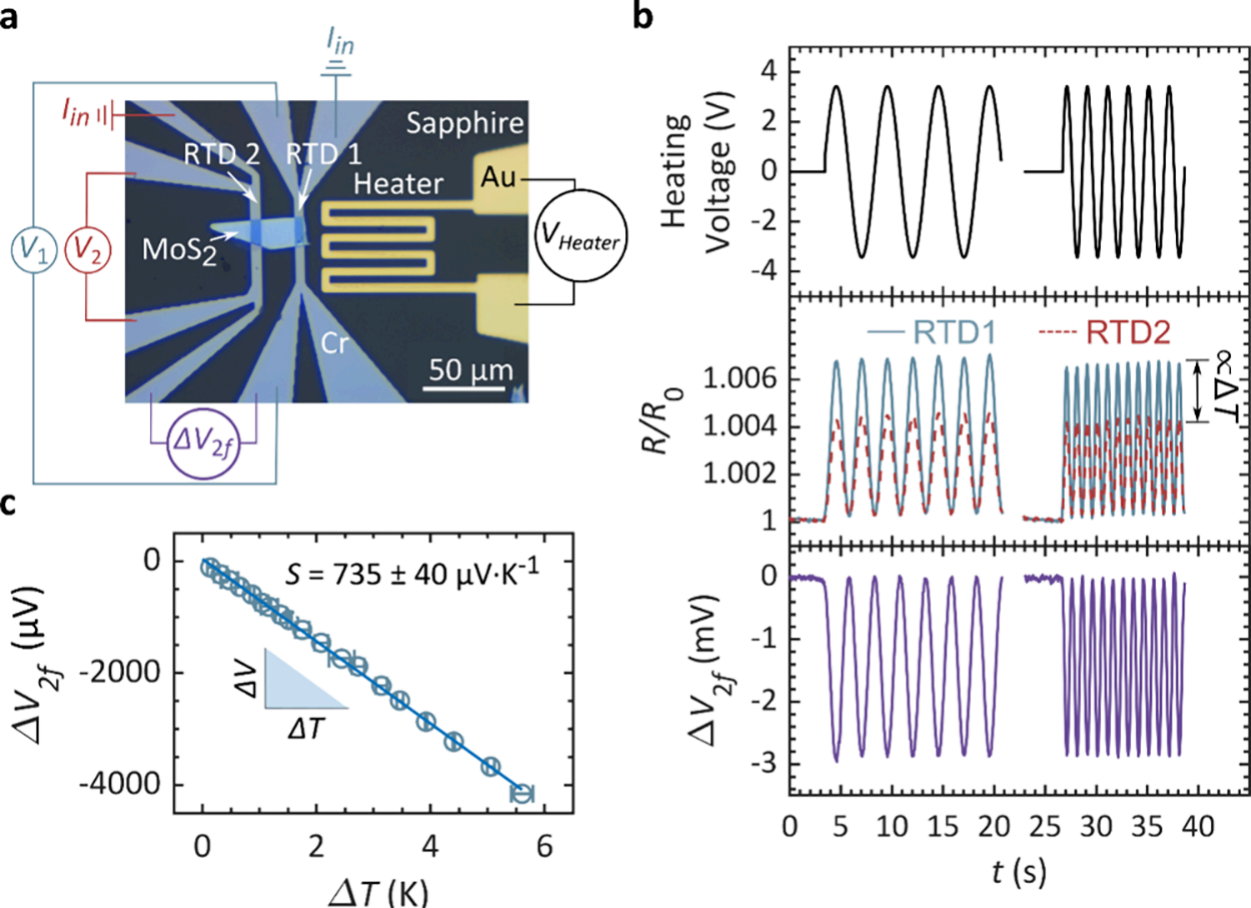
Thermoelectric measurement of MoS_2_ at room temperature. (a) Optical micrograph of a device consisting of a MoS_2_ crystal contacted by two electrodes that are used as RTDs and a heater on a sapphire substrate. (b) Top: sinusoidal input heating voltage with two different frequencies *f* (0.2 Hz on the left and 0.5 Hz on the right) with an amplitude of 3.2 V. Centre: relative change of the resistance measured for each RTD. The resistance change of each RTD is proportional to its temperature. The difference in gradients is proportional to the temperature gradient. Bottom: voltage induced in the MoS_2_ crystal (c) Thermoelectric voltage generated by the crystal as a function of the temperature gradient to which it is subjected. This thermoelectric corresponds to the pristine material (naturally n-type), and as expected, the Seebeck coefficient is negative.

For thermoelectric characterization, we fabricated devices comprising a heater and two resistive thermometers, as illustrated in Figure 3a. Two Pt leads serve as electrodes and resistance temperature detectors (RTD 1 and 2). An alternating current with frequency *f* is applied to the heater, generating a time-varying temperature gradient along the substrate. Simultaneously, we measure the resistances of the RTDs, as shown in Figure 3b. The resulting temperature variation occurs at twice the frequency of the heater voltage, consistent with the expected behavior of Joule heating. This approach, in combination with a previous calibration of the RTDs, enables to determine the temperature gradient Δ*T*_2f_ between the RTDs. Within the MoS_2_ crystal appears a thermally induced voltage in response to the temperature gradient at the frequency 2f. This voltage Δ*V*_2f_ can be directly measured, and thus, the in-plane Seebeck coefficient is determined with *S* = Δ*V*_2f_/Δ*T*_2f_ – *S*_metal_ (Figure 1d). For all experiments, we use low frequencies in the range of 0.2 to 0.5 Hz. In this range, the Δ*V*_2f_ amplitude is constant, as shown in the bottom panel of Figure 3b, which indicates that the temperature can be readily modulated. We choose this range because at higher frequencies (in the range of a few Hz), we find that the Δ*V*_2f_ amplitude is decreased, which indicates that at higher frequencies, the Seebeck coefficient cannot be accurately estimated. Further details on the measurements can be found in the Supporting Information.

**Figure 4:**
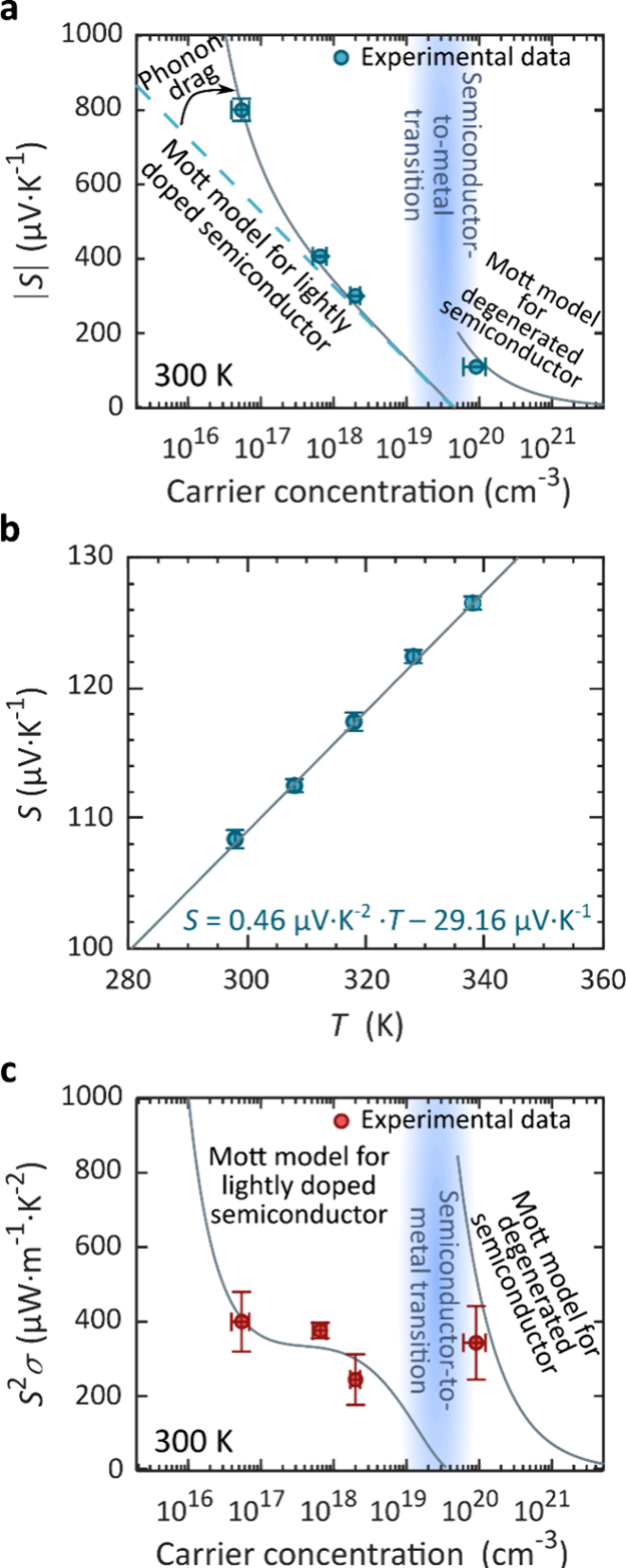
Carrier concentration dependence of the Seebeck coefficient and power factor. (a) Absolute value of the measured (dots) and modeled (line) Seebeck coefficient as a function of the carrier concentration. The value for ∼10^16^ cm^–3^ corresponds to pristine MoS_2_ (n-type), which is a negative value of the Seebeck coefficient. The three other values of higher carrier concentration (10^17^–10^20^ cm^–3^) correspond to p-type MoS_2_, and the Seebeck coefficient is positive. (b) Seebeck coefficient against temperature for the highly p-type-doped crystal showing a linear fit corresponding to the Mott formula for degenerate semiconductors. (c) Power factor against carrier concentration.

To compare the in-plane Seebeck coefficients as a function of doping concentration, we have used the same material from four batches as in the Hall experiments. Thin crystals of thicknesses between 12 and 26 nm were exfoliated and measured such as discussed above. Figure 4a shows the dependence of the in-plane Seebeck coefficient on the carrier concentration. The absolute value of the Seebeck coefficient is shown to accommodate positive and negative Seebeck coefficients. p-type MoS_2_ is found to be positive, which is consistent with p-type carrier transport, and pristine MoS_2_ crystals have a negative Seebeck coefficient (*n* ∼ 10^17^ cm^–3^). The Seebeck coefficient can be approximated using the Mott equation,^59^ which relates *S* to the logarithmic derivative of electrical conductivity,
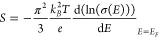
2where *T* is the temperature, *k*_B_ is the Boltzmann constant, and *E*_F_ is the Fermi energy. For an intrinsic or lightly doped semiconductor, the energy difference between the electrochemical potential and the occupied level governs the Seebeck coefficient.^60^ In this case, eq 2 can be written as^61^
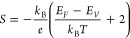
3where the constant 2 corresponds to scattering due to acoustic phonons and *E*_F_ – *E*_V_ is the energy difference between the Fermi level and the valence band edge. This difference can be estimated from the following expression, using the effective densities of states *N*_V_ reported in ref (62) assuming a degeneracy of 1 in the valence band:^63^
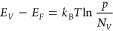
4

The result is shown with a dotted line in Figure 4a. Note that it has no fitting parameters. At low concentrations, this simple model underestimates the Seebeck coefficient, which could be attributed to phonon drag^64^ that gives an additional term of  where the specific heat per mole (*c*_V_) times the volume per mole (ρ) for MoS_2_ is 1.93 J K^–1^ cm^–3^. The parameter *f*_d_ represents the fraction of the phonons that contribute to phonon drag and is used as the fitting parameter. Phonon drag occurs when a portion of the charge carrier momentum is transferred to the crystal lattice, specifically to phonons. The model including this term is plotted as a solid line in Figure 4a and agrees well with the data for lightly doped semiconductors. The fit suggests that the fraction *f*_drag_ = 10^–6^ of phonons could contribute to phonon drag. This term causes the nonlinearity of the Seebeck coefficient with the logarithm of the carrier concentration shown in Figure 4a.

Equation 4 implies that at concentrations above ∼5 × 10^19^ cm^–3^, our material is degenerate, which occurs when (*E*_V_ – *E*_F_) ≲ *k*_B_*T*. For concentrations exceeding this threshold, a phase transition from the semiconducting to the metallic state is observed, primarily attributed to a structural transformation from the semiconducting H-phase to the metallic T-phase due to hole injection.^65^ This agrees with previously published results, and accordingly, the Mott model for degenerated semiconductors and metals applies for higher concentrations^66^
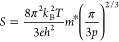
5

To apply this model in the range above the semiconductor-to-metal transition, we first determine the effective mass m* by measuring the temperature dependence of the Seebeck coefficient for *p* ≅ 10^20^ cm^–3^ (see Figure 4b). We observe an increase in the Seebeck coefficient with temperature, which is related to the enhanced scattering of carriers. From the slope, we determine effective mass as m* = (1.38 ± 0.03) *m*_0_, where *m*_0_ is the electron mass. Having determined m* permits us to represent the Mott model for degenerated semiconductors into Figure 4a, and we find good agreement to our data.

The trend of the Seebeck coefficient to reduce with increasing carrier concentration is the conventional trade-off in thermoelectrics: it is difficult to increase conductivity and Seebeck coefficient independently. When the carrier concentration increases, the Fermi energy approaches the valence band edge, and the differential conductivity becomes symmetric with respect to the Fermi energy. As the asymmetry decreases the Seebeck coefficient decreases. In terms of power factor, this often causes a bell-like curve in which the power factor has a local maximum and decreases for higher concentrations.^40,67^ Here, we find a convoluted dependency, possibly due to the presence of phonon drag, and the semiconductor-to-metal transition. To model the power factor, we assume that the transition from semiconductor to semimetallic takes place around 5 × 10^19^ cm^–3^ where both predictions diverge. Combining the calculations for the Seebeck coefficient (Figure 4a) and the linear fit in the conductivity vs carrier concentration (Figure 1d), we draw *S*^2^σ in Figure 4c as a prediction originated in the Mott model, and we find reasonable agreement with the experimental data. Below the semiconductor-to-metal transition, the model predicts the highest values at lowest concentrations and a decrease with increasing concentration with a plateau in the experimental range. Just above the transition, the values are predicted to be high, and a steep decrease is predicted. The precise behavior in this region is beyond the models used here. The maximum experimental value obtained is 400 ± 80 μW m^–1^ K^–2^, which corresponds to the least doped material (pristine), followed by the degenerate material with 340 ± 90 μW m^–1^ K^–2^. The Supporting Information contains a comparison of this result to literature values on other TMDs and two-dimensional layered materials.

**Figure 5:**
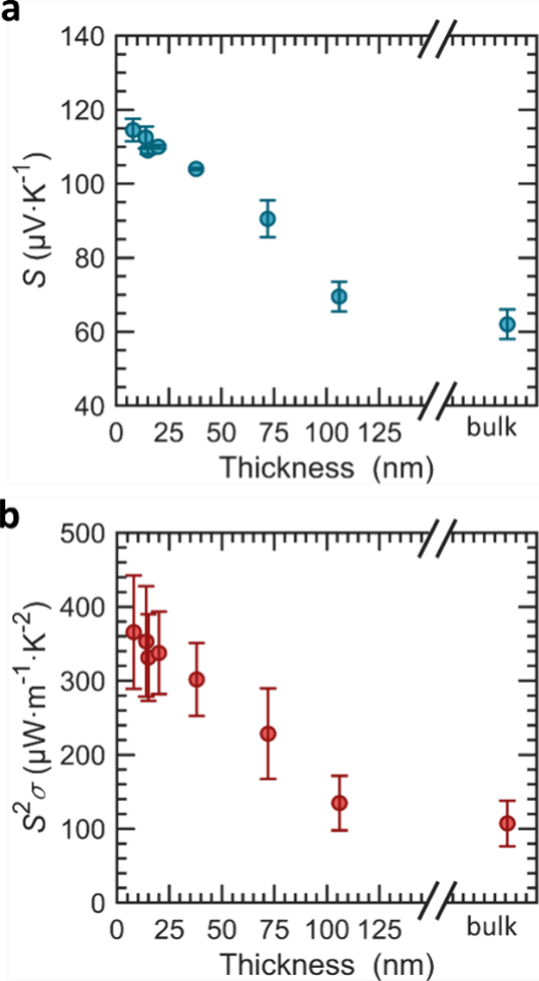
Thickness dependence of the Seebeck coefficient and power factor. (a) The Seebeck coefficient increases with decreasing crystal thickness. (b) The power factor increases with decreasing thickness since conductivity and Seebeck coefficient are decoupled.

Figure 5 shows the thermoelectric parameters of MoS_2_ with a concentration of ∼10^20^ cm^–3^ for various thicknesses. A striking result is an enhancement of the Seebeck coefficient with decreasing thickness. This is especially remarkable considering that the conductivity is constant in this range, as discussed above regarding Figures 1 and 2. This is not the expected behavior for bulk materials in which the Seebeck coefficient is independent of the thickness. We measured the bulk value to be 62 ± 4 μV K^–1^ by mounting a thick (>10 μm) crystal onto a heating/cooling stage (see the Supporting Information). A linear fit to our data lets us identify the start of the bulk region to be in the order of ∼130 nm. This is lower than what has been reported for pristine MoS_2_^52^ (whereby in this reference, electrical conductance instead of Seebeck coefficient was analyzed). The discrepancy may be due to the high doping of Nb that leads to a degeneracy in the material and introduces the Fermi level in the valence band, giving the material a semimetallic character. It has been shown that the exfoliated 3R material contains a higher amount of 1T-MoS_2_ metallic phase than the bulk case,^68^ which contributes to a partially metallic character of the exfoliated material. An increase in metallicity is responsible for the decrease in Seebeck coefficient with increasing thickness. As the number of stacked layers decreases, this effect becomes weaker, as the electronic structure tends toward a few-layer or monolayer structure. A slow transition from semimetallic to semiconducting material on a scale of tens of nanometers has also been reported for another semimetal, PtSe_2_.^69,70^ Since the Seebeck coefficient increases with decreasing thickness but the conductivity is constant (see discussion of Figure 1), the conventional electrical conductivity/Seebeck coefficient trade-off has been overcome. The Seebeck coefficient is improved independently. This causes the power factor to follow a similar trend to the Seebeck coefficient with a peak value of 370 ± 80 μW m^–1^ K^–2^ for a thickness of 8 nm, which is superior to all bulk TMDs including bulk intrinsic MoS_2_^71^ and MoS_2_/CNT heterofilms^15^ except for TiS_2_.^72^

## Conclusions

In summary, we observe a doping concentration-mediated enhancement of electric and thermoelectric properties of Nb-doped MoS_2_ across various thicknesses. Our experimental characterizations, coupled with theoretical modeling, reveal the role of depleted regions on p-type TMDs. Furthermore, we find that the conductivity MoS_2_:Nb can be much higher than other TMDs, underscoring its promising performance for electronic applications.

This substitutional doping also enables the use as a thermoelectric device and provides an approach toward bipolar thermoelectric devices. By controlling the doping concentration and crystal thickness, we can decouple the Seebeck coefficient from the electrical conductivity, overcoming the trade-off between the two. This results in a large power factor of ∼370 μW m^−1^ K^−2^ at room temperature. In addition, our crystals are nontoxic, flexible, and made of abundant elements. Another promising application of such controlled doping in MoS_2_ p-type is a photovoltaic device.^12^ Beyond MoS_2_, the Nb mediated p-type charge carrier is applicable to other TMDs, including WS_2_ and WSe.^73,74^ We anticipate that the availability of stable and controlled p-type doping in TMDs will open new possibilities for applications in microscale on-chip cooling and thermal sensors, bipolar junctions, and photovoltaics.

## Supporting Information Available

Device fabrication; geometrical corrections to van der Pauw measurements; characterization of thermoelectric devices; measurement of the Seebeck coefficient of a bulk crystal of MoS_2_; bibliographic review of the power factor in transition metal dichalcogenides (TMDs) (PDF)

## Abbreviations

AFM: atomic force microscopy
CNT: carbon nanotubes
RTD: resistance temperature detectors
TMDs: transition metal dichalcogenides
