# Supplementary material for: High Conductivity and Thermoelectric Power Factor in p-Type MoS2 Nanosheets
Source: ACS Appl Energy Mater. 2025 Feb 5;8(6):3500–8. doi: 10.1021/acsaem.4c02932 (PMC11938393; doi:10.1021/acsaem.4c02932)
Supplement: Supplementary file 1 — ae4c02932_si_001.pdf [file ae4c02932_si_001.pdf]

Supporting Information: High conductivity and thermoelectric power factor in p-type MoS_2 nanosheets

Inés Durán[1], Carlos Bueno-Blanco[1], Jorge Rodríguez-Muro[1], Mario Martinez[1], Elizabeth Champa-Bujaico[1], Patricia Cancho García[1], Der-Yuh Lin[2], Antonio Marti[1], Elisa Antolin[1], Simon A. Svatek*[1]*

1. Instituto de Energía Solar, Universidad Politécnica de Madrid, Avenida Complutense 30, 28040, Madrid, Spain

2. Department of Electronics Engineering, National Changhua University of Education, Changhua 50007, Taiwan

*e-mail: simon.svatek@upm.es, elisa.antolin@upm.es

Section I: Device fabrication

Al_2O_3 substrates were purchased from Ossila Ltd. and have a roughness of ~0.3 nm. The SiO_2 substrates were purchased from Siegert Wafer GmbH and consist of crystalline silicon (CZ) with a thickness of 525 μm and a 290 nm SiO_2 thermal oxide. Bulk MoS_2 were grown by us by the chemical transport method except for pristine and p-MoS_2 with a carrier concentration of ~10^{18} cm^{-3}, which were purchased from HQ Graphene.

Devices were created using standard microfabrication methods. Photolithography was performed using a maskless technique with a Smart Print UV (Microlight 3D SAS). To establish ohmic contact between the metal electrodes and the crystals, we use Cr for n-

type MoS_2 and Pt for p-type MoS_2. Under very high doping concentrations, around 10^{20} cm^{-3}, the crystal behaves as a semi-metal and makes ohmic contact with Cr, Au and Pt. We use thermal evaporation for Cr and Au, and sputter deposition for Pt.

To contact n-type MoS_2 deposited on top of metal leads we fabricated 10 nm Cr/ 30 nm Au/ 15 nm Cr contacts. The top Cr layer makes contact to the semiconductor. The Au layer in the center facilitates thermoelectric measurements as it has a higher resistance temperature coefficient than Cr. The bottom Cr layer promotes adhesion to the substrate. In Figure S1a, the current-voltage characteristic of a representative n-type device demonstrates the ohmic behavior when contacted with Cr. The inset image illustrates an example of a device designed to measure the Seebeck coefficient of a n-type crystal. The same ohmic behavior was demonstrated for p-type MoS_2 (Figure S1b) and highly doped MoS_2 crystals (p^+-MoS_2) with a concentration above 10^{20} cm^{-3} (Figure S1c), which were contacted with 10 nm Cr / 40 nm Pt and 10 nm Cr / 40 nm Au, respectively.

The crystals were exfoliated on PDMS using adhesive tape (BT-150E-CM, Nitto) and selected based on shape, thickness, and uniformity using an optical microscope and AFM. To deposit the material between both electrodes, we used the hot pick-up technique[1].

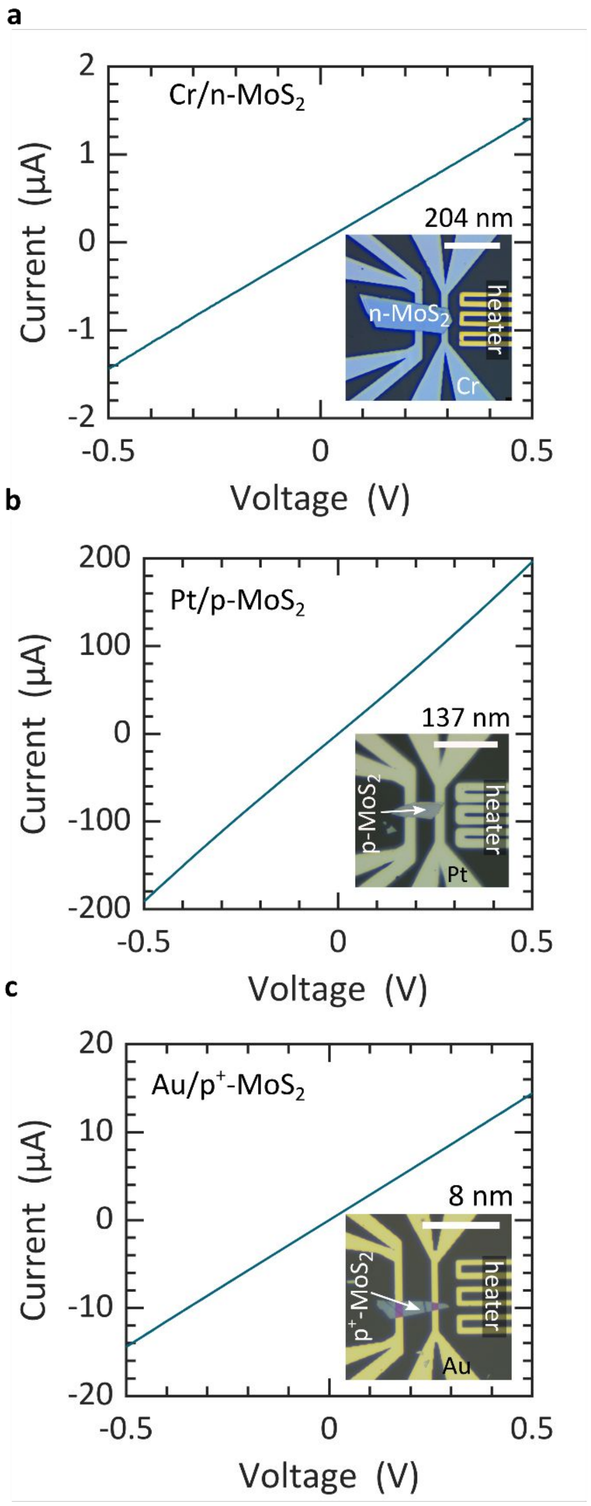

Figure S1. Current-voltage characteristic of (a) n-MoS2 crystal (204 nm) contacted with Pt, (b) p-MoS2 crystal (137 nm) contacted with Cr and (c) highly p-doped MoS2 crystal (p+-MoS2) (8 nm) contacted with Au. Insets show the images of the devices. The scale

bar corresponds to 50 μm and the crystal thickness is indicated above the photograph. The substrate is Al_2O_3 in all cases.

Section II: Geometrical corrections to Van-der-Pauw measurements

Van-der-Pauw measurements were performed using a source measure unit (Keithley 6221) as the current source and a high-precision voltmeter (Keithley 2182A) to measure voltages. To measure the sheet conductance, four-probe measurements were made where current was injected between two adjacent contacts and voltages were measured across the opposite contact pair. To measure the Hall resistance, we injected current between two opposite contacts and measured the voltage across the other two contacts using a magnetic field of 0.55 T. To deduce the sheet conductance, sheet carrier density, and mobility, we used the standard van der Pauw formulas[2]. To improve accuracy each measurement was performed with positive and negative current-flow and averaged. The error bars presented in the conductivity, depleted layer thickness, and mobility graphs represent the statistical error of the measurements.

The analysis of van-der-Pauw measurements assumes that the contacts are point-like and they are located at the edge of the crystal. However, in real experiments, these ideal conditions cannot be met. Therefore, it was necessary to apply geometric corrections to account for the non-ideality of the contacts[2–4]. In our case, both the contacts and the crystal are approximately square as shown in Figure S2. The contact area can be parametrized by the length c and L is the length of the crystal. For an ideal contact $c/L \rightarrow \infty$.

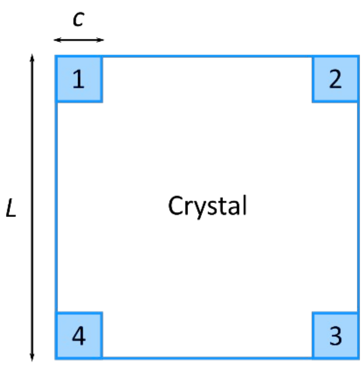

Figure S2. Sample contact geometry schematic showing finite square contact sizes of width c, used for van-der-Pauw measurements. We consider that the crystal under study is also considered to be a square with a width L.

We denote $R_{\square}$ the sheet resistance measured using the van-der-Pauw ideal method. A correction factor $\Delta R_{\square\mathrm{i}}$ can be applied for each contact, written as[3]:

$$\Delta R_{\square\mathrm{i}} = f_{\rho,i}\left(\frac{c_i}{L}\right)^2, \qquad \text{(S1)}$$

where $f_{\rho,i}$ is the resistivity correction factor for the contact i (where i goes from 1 to 4 denoting each of the four contacts) and it is a function of $(c_i/L)^2$. By averaging the four contacts we obtain:

$$R_{\square,corrected} \cong R_{\square} + \hat{f}_{\rho}\frac{\sum_{i=1}^{4}\left(\frac{c_i}{L}\right)^2}{4}. \qquad \text{(S2)}$$

Where we use a single factor $\hat{f}_{\rho}$ that corresponds to the average of the $(c_i/L)^2$ terms. In this process, while analyzing the same MoS_2 material, we obtained a corrected resistivity value for each crystal.

To calculate the electronic mobility μ and carrier concentration n, the Hall resistance R_{Hall} was measured. The non-ideality of the contacts also affects the accuracy of this

measurement, requiring for each contact an additional correction term to be applied, which was defined as[3]:

$$\Delta R_{Hall,i} = f_{Hall,i}\left(\frac{c_i}{L}\right), \quad \text{(S3)}$$

where $f_{Hall,i}$ is the Hall voltage correction factor for the contact i (where i goes from 1 to 4 denoting each of the four contacts) and it is a function of (c_i/L). The corrected Hall resistance considering all the contacts was calculated as:

$$R_{Hall,corrected} = R_{Hall} + \sum_{i=1}^{4} \Delta R_{Hall,i}. \quad \text{(S4)}$$

The 2D carrier concentration n_{2D} and the electron mobility μ were then calculated using the following expressions:

$$n_{2D} = \frac{B}{R_{Hall,corrected} \cdot e}, \quad \text{(S5)}$$

$$\mu = \frac{1}{e \cdot n_{2D} \cdot R_{\square,corrected}} \quad \text{(S6)}$$

Section III: Characterization of the thermoelectric devices

For thermoelectric characterization, we first calibrated the resistance temperature detectors (RTD_1 and RTD_2) which also act as electrodes contacting the MoS_2 crystals. The resistance of both thermometers, R_1 and R_2, are individually measured using four probes with a source-measure unit (Keysight B2901A) and a laboratory hotplate to control their temperature. While modifying the temperature of the hotplate we measured the current flowing through both thermometers ($I_1(T)$ and $I_2(T)$) when the voltage is set to a certain value, V_1 and V_2 (we chose the same voltage for both thermometers). We then calculated the resistance of each thermometer for each temperature. From plots of the resistance of each thermometer, R_1 and R_2, as a function of the temperature we determine

the slope (m_{RT}) which corresponds to the change of resistance with temperature [Ω/K], as shown in Figure S3a.

$$m_{RT} = \frac{\Delta R}{\Delta T} \quad \text{(S7)}$$

We estimate the temperature coefficient of resistance α in $°C^{-1}$, which describes its relative change for a given change in temperature, as

$$R = R_0(1 + \alpha(T - T_0)) \quad \text{(S8)}$$

where R_0 and T_0 are the resistance and the reference temperature, respectively. From (S7) and (S8), we can calculate

$$\alpha = \frac{m_{RT}}{R_0} \quad \text{(S9)}$$

Once the thermometers are calibrated, we apply a sinusoidal heating voltage of frequency f and amplitude $V_{0,\mathrm{H}}$ to the heater, $V_{\mathrm{H}}(f)$, with a function generator (Tektronix, model AFG3022B) as shown in Figure S3b. The heater generates a power given by

$$P_H = \frac{V_H^2}{R_H} \propto V_H^2 \quad \text{(S10)}$$

where R_H is the resistance of the heater, calculated from *I-V* characteristics. This heating power is proportional to the square of the voltage applied to the heater, oscillating in consequence with a frequency of $2f$. This heat induces a periodic temperature gradient with an amplitude ΔT_{2f} at the same frequency $2f$, which appears between the thermometers causing a periodic thermally induced voltage in the crystal with an amplitude ΔV_{2f}. This amplitude is directly measured using a lock-in amplifier (Stanford Research Systems, model SR830 DSP), as schematically represented in Figure S3c. The sign of ΔV_{2f} indicates the type of doping: negative for n-type and positive for p-type.

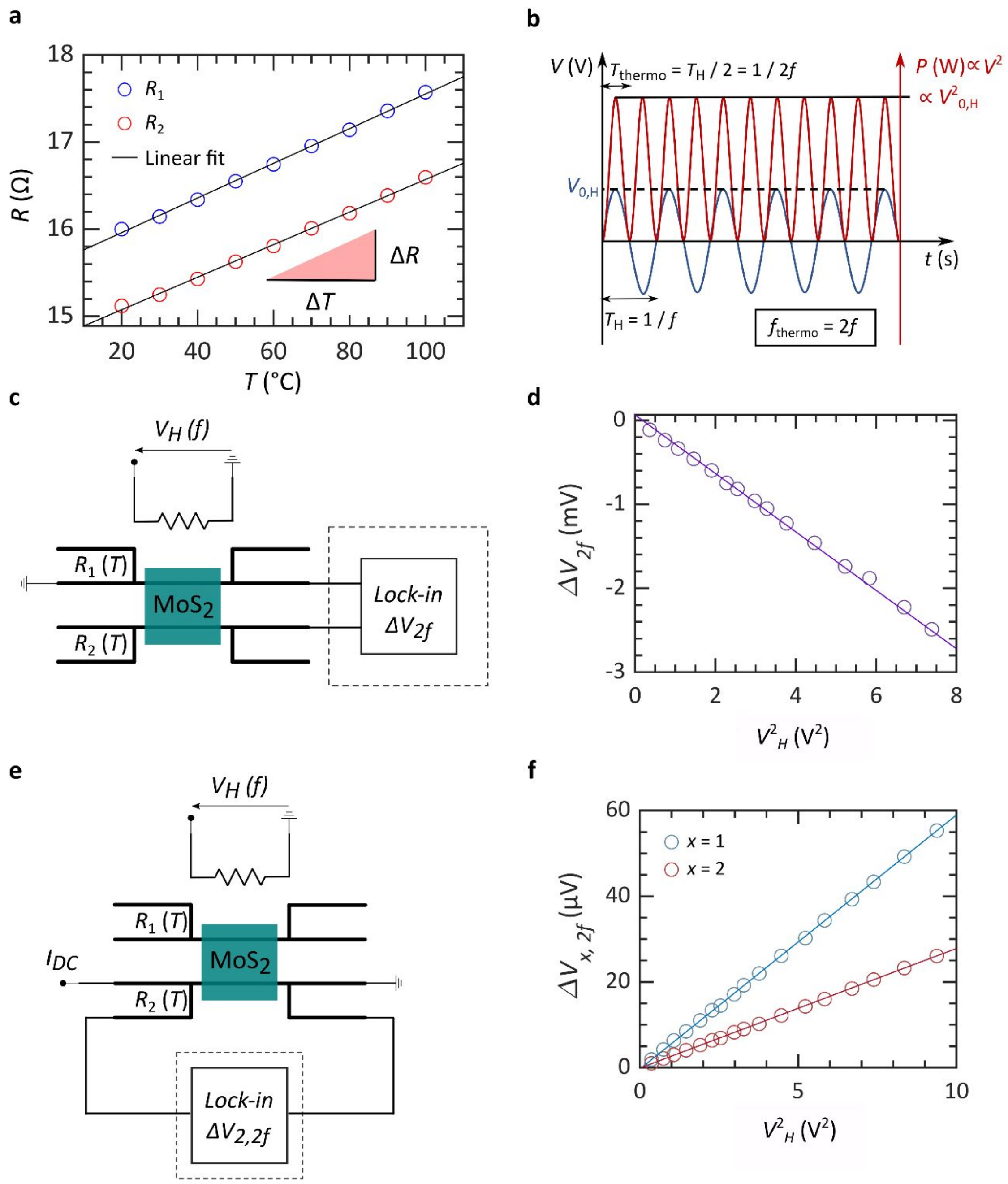

Figure S3. Thermoelectric characterization panel. (a) Resistance of RTD1 and RTD2, R_1 and R_2, respectively, as a function of temperature. (b) Schematic indicating the shape of the signal applied to the heater with a frequency f and an amplitude $V_{0,\mathrm{H}}$ and how it translates into a heating power with an amplitude proportional to $V^2_{0,\mathrm{H}}$ and a frequency 2f. (c) Measurement of thermally induced voltage across the crystal. (d) Voltage induced in the crystal as a function of V^2_{H}. (e) Measurement of the temperature gradient in the crystal. (f) Temperature gradient induced in each electrode (x) as a function of V^2_{H}.

To measure the amplitude of the temperature gradient between the thermometers (RTD 1 and RTD 2), ΔT_{2f}, we measure separately the amplitude of the temperature gradient generated in each of them ($\Delta T_{1,\,2f}$ and $\Delta T_{2,\,2f}$, respectively). To determine $\Delta T_{x,\,2f}$ (where x = 1 or 2), we apply a direct current (I_{DC} ~ 10 μA) and measure the variation of voltage within the electrodes due to the variation of resistance. The change in voltage across the RTD is measured using the same lock-in amplifier at a reference frequency of $2f$ ($\Delta V_{x,\,2f}$), as illustrated in Figure S3e. The change of resistance in each electrode, $\Delta R_{x,\,2f}$, is

$$\Delta R_{x,\,2f} = \frac{I_{\mathrm{DC}}}{\Delta V_{x,\,2f}} \quad \text{(S11)}$$

and, consequently, the change in the temperature of each electrode is calculated from (S8) with:

$$\Delta R_{x,\,2f} = R_{x,\,hot} - R_{x,cold}$$

$$\Delta R_{x,\,2f} = R_0(1 + \alpha(T_{x,\,hot} - T_0)) - R_0(1 + \alpha(T_{x,cold} - T_0))$$

$$\Delta R_{x,\,2f} = R_0(1 + \alpha(T_{x,\,hot} - T_{x,cold}))$$

$$\Delta R_{x,\,2f} = R_0\big(1 + \alpha(\Delta T_{x,\,2f})\big)$$

$$\Delta T_{x,\,2f} = \frac{\left(\frac{\Delta R_{x,\,2f}}{R_0} - 1\right)}{\alpha} \quad \text{(S12)}$$

The temperature gradient induced in each RTD as a function of V_H^2 is plotted for an example device in Figure S3f. The total voltage appearing at the source meter (ΔV_{2f}) per temperature gradient is the sum of the Seebeck coefficients of the MoS_2 (S) and the metal leads, and therefore

$$S = \frac{\Delta V_{2f}}{\Delta T_{2f}} - S_{metal}, \quad \text{(S13)}$$

where S_{metal} is the Seebeck coefficient of the electrodes (S_{Au} ~6.5 μV/K, S_{Pt} = 0 μV/K, S_{Cr}=18 [5]).

Section IV: Measurement of the Seebeck coefficient of a bulk crystal of MoS_2

To measure the bulk value of the Seebeck coefficient, we set up a different experiment because our micrometer-sized electrodes give poor control when used with large crystals (>1 mm) with thicknesses of many micrometers. We mount the crystal into a printed circuit board (PCB) and cool one end with a Peltier temperature-controller breadboard (Thorlabs, model PTC1/M), while heating the other end with a metal ceramic heater (Thorlabs, HT24S - 24 W). This creates a constant temperature gradient that is determined using two thermocouples. This measuring set-up is shown in Figure S4a.

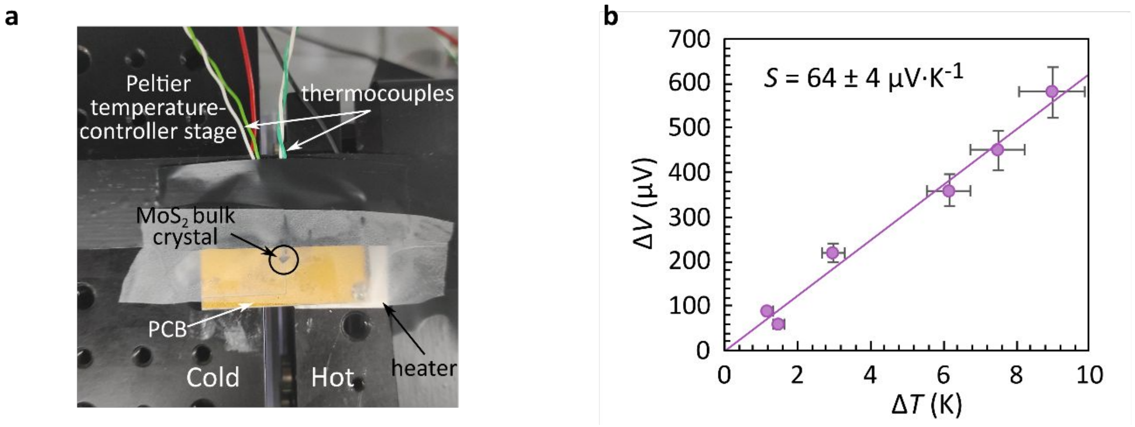

Figure S4. Measurement of the Seebeck coefficient of a bulk crystal of MoS_2. (a) Image of the experimental set-up designed to measure the Seebeck coefficient of a macroscopic crystal. (b) Voltage induced in the bulk crystal as a function of temperature gradient.

By adjusting the power applied to the heater, we vary the temperature gradient while measuring the induced voltage. Figure S4b shows this voltage as a function of the temperature gradient. Using (14) we determine $S = (64 \pm 4\ \mu V \cdot K^{-1})$ from the slope of the linear fit of the experimental data. The thickness of this crystal was measured with a

profilometer (KLA Tencor, model AlphaStep D-120) and was found to be between 20 and 70 μm.

Section V: Bibliographic review of the power factor in Transition Metal Dichalcogenides (TMDs)

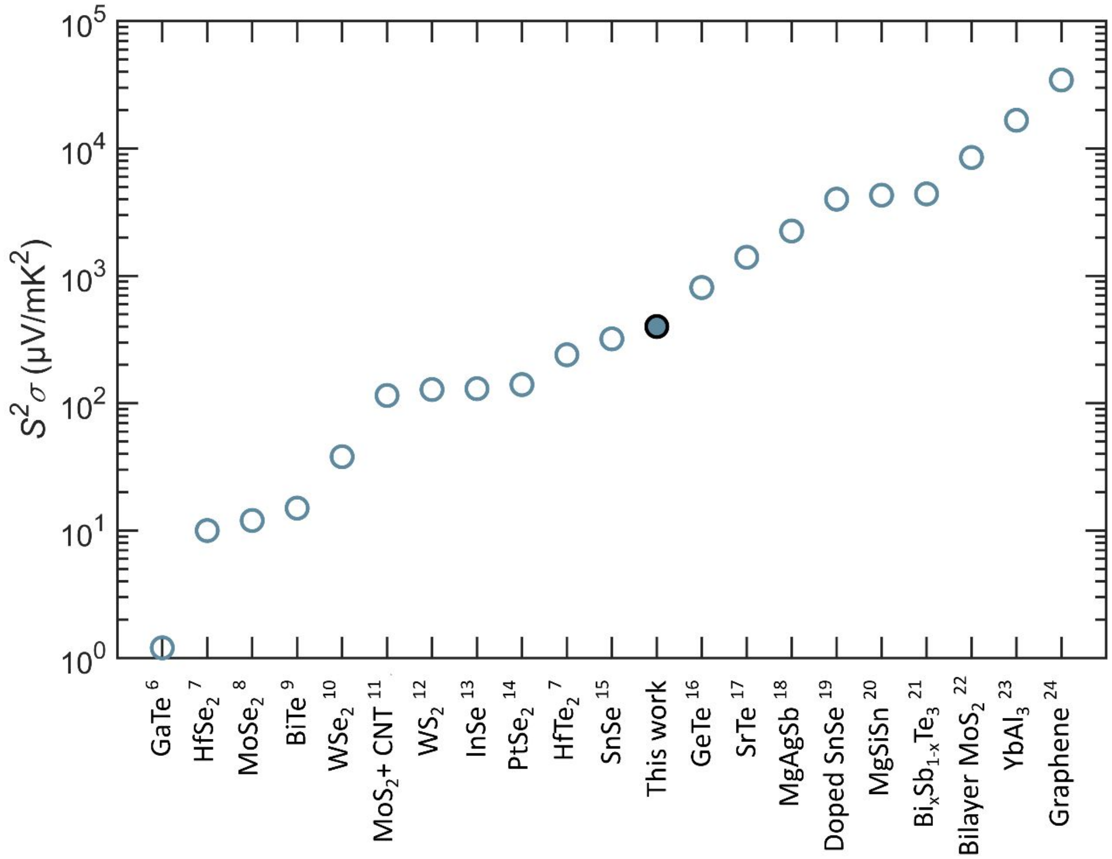

Figure S5. Graph displaying a set of power factor values collected from the literature for various TMDs and other two-dimensional layered materials. Each point represents a material, labeled on the x-axis alongside its corresponding bibliographic reference. The shaded point indicates the power factor result presented in this work.

References

(1) Pizzocchero, F.; Gammelgaard, L.; Jessen, B. S.; Caridad, J. M.; Wang, L.; Hone, J.; Bøggild, P.; Booth, T. J. The Hot Pick-up Technique for Batch Assembly of van Der Waals Heterostructures. *Nat. Commun.* **2016**, *7* (1), 11894.
(2) van der Pauw, L. J. A Method of Measuring Specific Resistivity and Hall Effect of Discs of Arbitrary Shape. *Philips Res. Rep* **1958**, *13* (1), 1–9.
(3) Look, D. C. Electrical Characterization of GaAs Materials and Devices. *Wiley* **1989**.

(4) Lin, Y.-J.; Su, T.-H. SiO_2 Substrate Passivation Effects on the Temperature-Dependent Electrical Properties of MoS_2 Prepared by the Chemical Vapor Deposition Method. *J. Mater. Sci.: Mater. Electron* **2017**, *28* (14), 10106–10111. https://doi.org/10.1007/s10854-017-6772-2.

(5) Arajs, S.; Anderson, E. E.; Rao, K. V. Seebeck Coefficient of Binary Chromium Alloys Containing Molybdenum or Tungsten. *J. Less-Common Met.* **1972**, *26* (1), 157–164.

(6) Vu, T. H.; Pham, A. T.; Park, J.; Park, S.; Cho, S. Bi-Doped GaTe Single Crystals: Growth and Thermoelectric Properties. *J. Solid State Chem.* **2021**, *298*, 122155.

(7) Bang, J.; Kim, H.-S.; Kim, D. H.; Lee, S. W.; Park, O.; Kim, S. Phase Formation Behavior and Electronic Transport Properties of $HfSe_2$-$HfTe_2$ Solid Solution System. *J. Alloys Compd.* **2022**, *920*, 166028.

(8) Kim, H. J.; Van Quang, N.; Nguyen, T. H.; Kim, S.; Lee, Y.; Lee, I. H.; Cho, S.; Seong, M.-J.; Kim, K.; Chang, Y. J. Tuning of Thermoelectric Properties of $MoSe_2$ Thin Films Under Helium Ion Irradiation. *Nanoscale Res. Lett.* **2022**, *17* (1), 26. https://doi.org/10.1186/s11671-022-03665-9.

(9) Qin, D.; Pan, F.; Zhou, J.; Xu, Z.; Deng, Y. High ZT and Performance Controllable Thermoelectric Devices Based on Electrically Gated Bismuth Telluride Thin Films. *Nano Energy* **2021**, *89*, 106472.

(10) Yoshida, M.; Iizuka, T.; Saito, Y.; Onga, M.; Suzuki, R.; Zhang, Y.; Iwasa, Y.; Shimizu, S. Gate-Optimized Thermoelectric Power Factor in Ultrathin WSe_2 Single Crystals. *Nano Lett.* **2016**, *16* (3), 2061–2065. https://doi.org/10.1021/acs.nanolett.6b00075.

(11) Li, J.; Shi, Q.; Röhr, J. A.; Wu, H.; Wu, B.; Guo, Y.; Zhang, Q.; Hou, C.; Li, Y.; Wang, H. Flexible 3D Porous MoS_2/CNTs Architectures with *ZT* of 0.17 at Room Temperature for Wearable Thermoelectric Applications. *Adv. Funct. Mater.* **2020**, *30* (36), 2002508. https://doi.org/10.1002/adfm.202002508.

(12) Kim, J. H.; Yu, S.; Lee, S. W.; Lee, S.-Y.; Kim, K. S.; Kim, Y. A.; Yang, C.-M. Enhanced Thermoelectric Properties of WS_2/Single-Walled Carbon Nanohorn Nanocomposites. *Crystals* **2020**, *10* (2), 140.

(13) Lee, S. W.; Kim, T.; Kim, H.-S.; Park, O.; Kim, D. H.; Kim, S. Enhanced Thermoelectric Properties of InSe through Simultaneous Increase in Electrical Conductivity and Seebeck Coefficient by Cl Doping. *J. Mater. Res. Technol.* **2022**, *19*, 2077–2083.

(14) Moon, H.; Bang, J.; Hong, S.; Kim, G.; Roh, J. W.; Kim, J.; Lee, W. Strong Thermopower Enhancement and Tunable Power Factor *via* Semimetal to Semiconductor Transition in a Transition-Metal Dichalcogenide. *ACS Nano* **2019**, *13* (11), 13317–13324. https://doi.org/10.1021/acsnano.9b06523.

(15) Zhao, L.-D.; Lo, S.-H.; Zhang, Y.; Sun, H.; Tan, G.; Uher, C.; Wolverton, C.; Dravid, V. P.; Kanatzidis, M. G. Ultralow Thermal Conductivity and High Thermoelectric Figure of Merit in SnSe Crystals. *Nature* **2014**, *508* (7496), 373–377.

(16) Placheova, S. K. Thermoelectric Figure of Merit of the System $(GeTe)_{1-x}(AgSbTe_2)_x$. *Phys. Stat. Sol. (a)* **1984**, *83* (1), 349–355. https://doi.org/10.1002/pssa.2210830140.

(17) Biswas, K.; He, J.; Blum, I. D.; Wu, C.-I.; Hogan, T. P.; Seidman, D. N.; Dravid, V. P.; Kanatzidis, M. G. High-Performance Bulk Thermoelectrics with All-Scale Hierarchical Architectures. *Nature* **2012**, *489* (7416), 414–418.

(18) Zhao, H.; Sui, J.; Tang, Z.; Lan, Y.; Jie, Q.; Kraemer, D.; McEnaney, K.; Guloy, A.; Chen, G.; Ren, Z. High Thermoelectric Performance of MgAgSb-Based Materials. *Nano Energy* **2014**, *7*, 97–103.

(19) Zhao, L.-D.; Tan, G.; Hao, S.; He, J.; Pei, Y.; Chi, H.; Wang, H.; Gong, S.; Xu, H.; Dravid, V. P.; Uher, C.; Snyder, G. J.; Wolverton, C.; Kanatzidis, M. G. Ultrahigh Power Factor and Thermoelectric Performance in Hole-Doped Single-Crystal SnSe. *Science* **2016**, *351* (6269), 141–144. https://doi.org/10.1126/science.aad3749.

(20) Liu, W.; Tan, X.; Yin, K.; Liu, H.; Tang, X.; Shi, J.; Zhang, Q.; Uher, C. Convergence of Conduction Bands as a Means of Enhancing Thermoelectric Performance of N-Type $Mg_2Si_{1-x}Sn_x$ Solid Solutions. *Phys. Rev. Lett.* **2012**, *108* (16), 166601. https://doi.org/10.1103/PhysRevLett.108.166601.

(21) Kim, S. I.; Lee, K. H.; Mun, H. A.; Kim, H. S.; Hwang, S. W.; Roh, J. W.; Yang, D. J.; Shin, W. H.; Li, X. S.; Lee, Y. H.; Snyder, G. J.; Kim, S. W. Dense Dislocation Arrays Embedded in Grain Boundaries for High-Performance Bulk Thermoelectrics. *Science* **2015**, *348* (6230), 109–114. https://doi.org/10.1126/science.aaa4166.

(22) Hippalgaonkar, K.; Wang, Y.; Ye, Y.; Qiu, D. Y.; Zhu, H.; Wang, Y.; Moore, J.; Louie, S. G.; Zhang, X. High Thermoelectric Power Factor in Two-Dimensional Crystals of MoS_2. *Phys. Rev. B* **2017**, *95* (11), 115407.

(23) Rowe, D. M.; Kuznetsov, V. L.; Kuznetsova, L. A.; Min, G. Electrical and Thermal Transport Properties of Intermediate-Valence $YbAl_3$. *J. Phys. D: Appl. Phys.* **2002**, *35* (17), 2183.

(24) Duan, J.; Wang, X.; Lai, X.; Li, G.; Watanabe, K.; Taniguchi, T.; Zebarjadi, M.; Andrei, E. Y. High Thermoelectric Power Factor in Graphene/*h*-BN Devices. *Proc. Natl. Acad. Sci. U.S.A.* **2016**, *113* (50), 14272–14276.
